# Supplementary material for: Alteration in sB7-H4 Serum Levels and Placental Biomarker Expression after Therapeutic Plasma Exchange in Early-Onset Preeclampsia Patients
Source: Int J Mol Sci. 2024 Oct 15;25(20):11082. doi: 10.3390/ijms252011082 (PMC11507903; doi:10.3390/ijms252011082)
Supplement: Supplementary file 1 [file ijms-25-11082-s001.zip › ijms-3238579-supplementary.pdf]

Supplementary material: Duan, Ma et al.

Supplementary Table S1. Antibodies used for immunoblotting

Antigen	Species raised in	Supplier (Cat. No.)	Dilution
**Primary antibody**			
Flt-1	Rabbit	Santa Cruz Biotechnology (sc-316)	1:200
Endoglin	Goat	R & D systems (AF1097)	1:1000
B7-H4	Rabbit	R & D systems (#2318A)	1:1000
TNF-α	Rabbit	Abcam (ab183218)	1:1000
iNOS	Rabbit	Santa Cruz Biotechnology (sc-651)	1:250
β-Actin	Mouse	Sigma(A3854)	1:200,000
**Secondary antibody**			
rabbit, HRP	Goat	Invitrogen (G21234)	1:5000
Goat, HRP	Donkey	Invitrogen (A15999)	1:5000

Supplementary Table S2. Antibodies used for IF staining

Antigen	Species raised in	Supplier (Cat. No.)	Dilution
**Primary antibody**			
Flt-1	Rabbit	Santa Cruz Biotechnology (sc-316)	1:40
Endoglin	Goat	R & D systems (AF1097)	1:50
B7-H4	Rabbit	R & D systems (#2318A)	1:20
iNOS	Rabbit	Santa Cruz Biotechnology (sc-651)	1:25
**Secondary antibody**			
Rabbit-Alexa 488	Donkey	Abcam (ab150073)	1:200
Mouse-Cy3	Goat	Life Technologies (A10521)	1:200

Goat-Alexa 488	Donkey	Life Technologies (A11055)	1:200
Rabbit, HRP	Goat	Invitrogen (G21234)	1:100

Supplementary Table S3. ELISA kits

ELISA Kit	Detection Limit	Intra-assay % CV	Inter-assay % CV
sFlt-1 (BRAHMS sFlt-1 KRYPTOR assay, Cat. No. 845.075)	22 pg/ml	<10%	<5%
PlGF (BRAHMS PlGF plus KRYPTOR assay, Cat. No. 859.075),	3.6 pg/ml	<10%	<5%
sEng (R&D Systems, Cat. No. DNDG00)	0.03 ng/ml	<4%	<7%
sB7-H4 (Cloud-Clone Corp, Cat. No. L211008816)	0.055 ng/ml	<10%	<12%

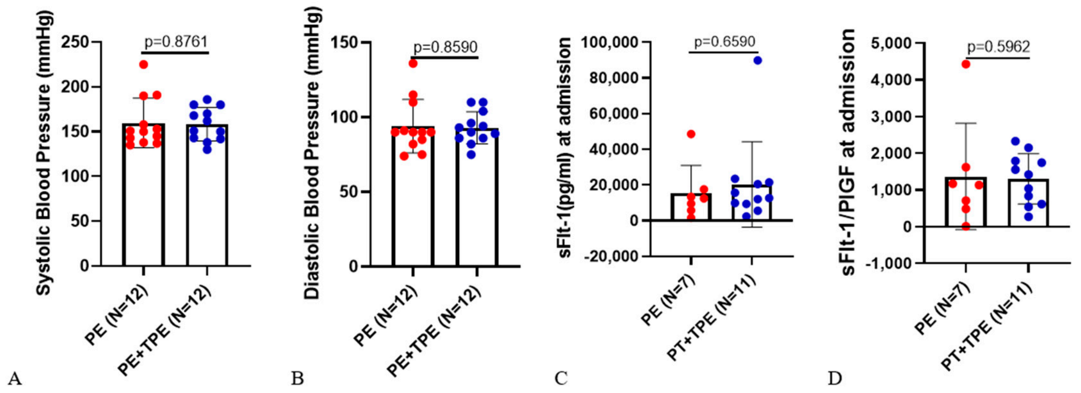

Supplementary Figure S1. Systolic blood pressure (A), diastolic blood pressure (B), sFlt-1 (C) and sFlt-1/PlGF (D) levels in PE and PE+TPE patients at admission. Data represent means ± SD.
